# A Novel Multi-Aperture Based Sun Sensor Based on a Fast Multi-Point MEANSHIFT (FMMS) Algorithm

**DOI:** 10.3390/s110302857

**Published:** 2011-03-03

**Authors:** Zheng You, Jian Sun, Fei Xing, Gao-Fei Zhang

**Affiliations:** 1 MOE Key Laboratory for Strength & Vibration, School of Aerospace, Xi’an Jiaotong University, Xi’an 710049, China; 2 State Key Laboratory of Precision Measurement Technology and Instruments, Department of Precision Instruments and Mechanology, Tsinghua University, Beijing 100084, China; E-Mails: yz-dpi@mail.tsinghua.edu.cn (Z.Y.); xingf02@mails.tsinghua.edu.cn (F.X.); zgf@mail.tsinghua.edu.cn (G.-F.Z.)

**Keywords:** APS sun sensor, multi-point MEANSHIFT algorithm, multi-aperture

## Abstract

With the current increased widespread interest in the development and applications of micro/nanosatellites, it was found that we needed to design a small high accuracy satellite attitude determination system, because the star trackers widely used in large satellites are large and heavy, and therefore not suitable for installation on micro/nanosatellites. A Sun sensor + magnetometer is proven to be a better alternative, but the conventional sun sensor has low accuracy, and cannot meet the requirements of the attitude determination systems of micro/nanosatellites, so the development of a small high accuracy sun sensor with high reliability is very significant. This paper presents a multi-aperture based sun sensor, which is composed of a micro-electro-mechanical system (MEMS) mask with 36 apertures and an active pixels sensor (APS) CMOS placed below the mask at a certain distance. A novel fast multi-point MEANSHIFT (FMMS) algorithm is proposed to improve the accuracy and reliability, the two key performance features, of an APS sun sensor. When the sunlight illuminates the sensor, a sun spot array image is formed on the APS detector. Then the sun angles can be derived by analyzing the aperture image location on the detector via the FMMS algorithm. With this system, the centroid accuracy of the sun image can reach 0.01 pixels, without increasing the weight and power consumption, even when some missing apertures and bad pixels appear on the detector due to aging of the devices and operation in a harsh space environment, while the pointing accuracy of the single-aperture sun sensor using the conventional correlation algorithm is only 0.05 pixels.

## 1. Introduction

The sun sensor is one of the most important devices for satellites. The early sun sensors were mainly sun appearance sensors and analog sun sensors [1]. With the development of space technology in recent years, requirements for sun sensors has been extended to a large field of view and high accuracy levels. The traditional design and implementation methods cannot meet these the requirements, so the single-axis digital sun sensor [2] appeared, with an accuracy of better than 0.1°. Two of the same single-axis digital sun sensors mounted vertically can constitute a conventional two-axis sun sensor. Such digital sun sensors are widely used currently.

**Figure 1.**
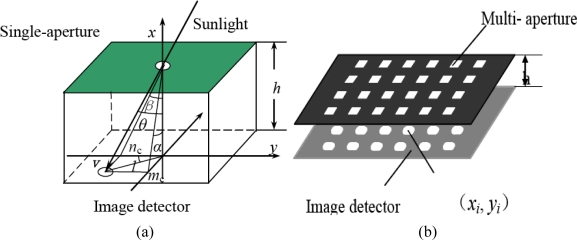
**(a)** single-aperture sun sensor **(b)** multi-aperture sun sensor.

Array image sensors such as CCD or CMOS and so on have been widely applied in space science. The array image sensor-based sun sensor is currently a research focus [3]. This kind of equipment is of high accuracy, high anti-interference. The aperture of sun sensors can be divided into single-aperture [as shown in Figure 1(a)] and multi-aperture [as shown in Figure 1(b)]. Compared with the traditional linear array sun sensor, the accuracy of a single-aperture sun sensor is increased, however, since only one spot is used in the system, image utilization rate is low, so it is difficult to further enhance its accuracy. The Italian Galileo Avionica Company has developed a single-aperture sun sensor, named Smart Sun Sensor [4].

Compared with the single-aperture sun sensor, a multi-aperture sun sensor has the following three advantages:

1. Accuracy: as the single-aperture sun sensor accuracy depends mainly on the positioning accuracy of the sun spot, and its pointing accuracy is only about 0.03° ,but for the multi-aperture sun sensor the accuracy of the sun spot location centroid is proportional to the square root of the number of apertures. Assuming that the pointing accuracy of a single hole *δ*, and hole arrays of *N* × *N*, then the final pointing accuracy is *δ/N*, This shows that multi-aperture can greatly improve the accuracy of the sun sensor.
2. Reliability: for the single-aperture sun sensor there is a very deadly danger: lack of reliability! When the hole is blocked or partially blocked, the accuracy will be greatly affected or even cause the system to lose function. For the multi-aperture sun sensor, the distribution of the image sensor area is relatively large, so even when part of the holes are completely blocked, using a reasonable method, the system still be able to guarantee the normal operation.
3. The image sensor requirements: the requirements of single-aperture sun sensor for image sensors are relatively high. The existence of bad pixels on the image sensor where the sun spot may fall will affect the accuracy and even function, while for the multi-aperture sun sensor, with a *N* × *N* imaging point, the distribution of the image sensor area is relatively large, the damage to individual pixels has almost no effect on the system accuracy.

From the above analysis we can find that multi-aperture sun sensors are superior to single-aperture sun sensors as far as the accuracy, reliability, and the requirements of the image sensor are concerned, so multi-aperture sun sensors must be the future direction of the technology.

## 2. Modeling of the Sun Sensor [5]

A multi-aperture sun sensor is commonly composed of a piece of mask and a CCD or APS (Active pixel sensor) CMOS detector below at a distance of about several millimeters. The sun spot is formed at the detector through the aperture, as shown in Figure 1(a). In the Figure 1(a), *m_c_* and *n_c_* denote the coordinates of the sun spot in the sun sensor body coordinates, *l* denotes the distance between the sun spot and hole sight point, *h* denotes the distance between the mask and the focal plane, *θ* denotes the incidence angle and *α*, *β* denote the sunray horizontal and azimuth orientation in the sun sensor body coordinates, respectively.

The relationships are as follows:

(1) $$\theta =\;\text{arctan}(\frac{l}{h}),\alpha =\;\text{arctan}(\frac{m_{c}}{h}),\beta =\;\text{arctan}(\frac{n_{c}}{h})$$

(2) $$l=\sqrt{m_{c}^{2}+n_{c}^{2}}$$

(3) $$\text{tan}\;\theta =\sqrt{{\left(\text{tan}\;\alpha \right)}^{2}+{\left(\text{tan}\;\beta \right)}^{2}}$$

**Figure 2.**
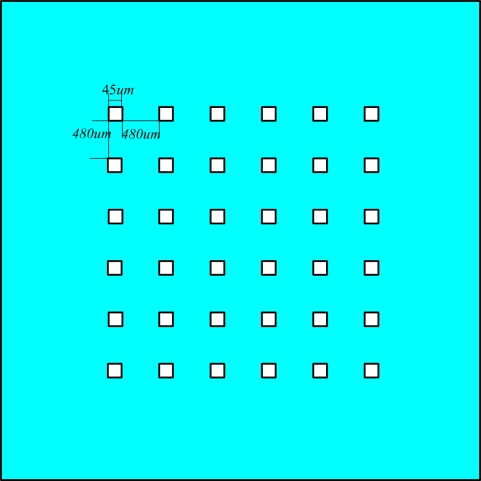
6 × 6 multi-aperture mask.

For a given distance *h*, the greater the number of apertures the higher the accuracy. However, the assembly and alignment errors will significantly impact the system accuracy when the location accuracy is of 1/100 pixels, about 0.005°. Generally, the sub-pixel accuracy of a single sun spot is at the level of 0.05–0.1 pixels, to obtain the whole sun spot location centroid accuracy of 1/100 pixels, the number of apertures should be more than 25 (1/100 = 0.05/251/2), so the aperture pattern is designed as a 6 × 6 array in this paper, as shown in Figure 2.

**Figure 3.**
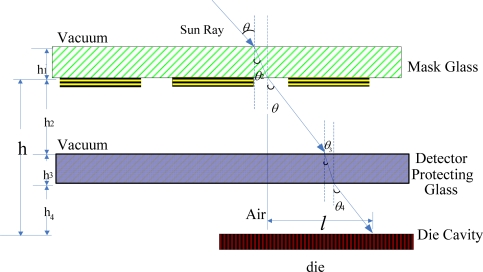
illustration of the sunray tracking route in the sun sensor.

Assuming the sun angles are *α,β* respectively and the sun spot centroid is *m_c_, n_c_* their relationship can be expressed by Equation (1). In fact, the Equation becomes much more complicated because of the refraction which occurs when a sun ray goes through different media sequentially (vacuum-mask glass-vacuum-detector protecting glass-air) before it arrives at the detector, as shown in Figure 3.

From Figure 3 we can conclude that the greater *h* the higher accuracy, however, the *h* must ensure that the image of sunlight in the field of view (FOV) is still within the photosensitive surface of the detector. So according to the effective detection area of the Star1000 and the sun sensor FOV design *h*, from the Figure 3, we can find *h* = *h_2_* + *h_3_* + *h_4_*; for the specific Star1000 *h_3_* and *h_4_* is fixed, when the image of sunlight in the FOV is within the photosensitive surface of the detector, the greater *h_2_* the higher accuracy. When the incident angle is large, *h_3_* and *h_4_* introduce nonlinear factors which may affect the sun sensor accuracy.

From Figure 3, we can conclude that the relationship between *l* and *θ* is:

(4) $$l=h_{2}\;\text{tan}\;\theta +h_{3}\;\text{tan}\;\theta_{3}+h_{4}\;\text{tan}\;\theta_{4}$$

(5) $$\frac{n_{\mathrm{Glass}}}{n_{\mathrm{Vacuum}}}=\frac{\text{sin}\;\theta }{\text{sin}\;\theta_{3}}$$

(6) $$\frac{n_{\mathrm{Air}}}{n_{\mathrm{Glass}}}=\frac{\text{sin}\;\theta_{3}}{\text{sin}\;\theta_{4}}$$

Where *n_glass_, n_vacuum_, n_air_* denote the light refractive index in the detector protecting glass, vacuum and air, respectively, and *l* is the displacement of the sun spot centroid in the case of normal incidence.

Substitution of *θ_3_* and *θ_4_* from Equations (5) and (6) into Equation (4) yields:

(7) $$l=h_{2}\;\text{tan}\;\theta +h_{3}\;\text{tan}(\text{arcsin}(\frac{\text{sin}\;\theta }{n_{\mathrm{Glass}}}))+h_{4}\;\text{tan}(\text{arcsin}(\frac{\text{sin}\;\theta }{n_{\mathrm{Air}}}))$$

Since *h_2_,h_3_,h_4_,n_glass_, n_air_* are all constants, *l* is a function of the incidence angle *θ*. Conversely, *θ* could be expressed as:

(8) $$\theta =f(l)=f(\sqrt{m_{c}^{2}+n_{c}^{2}})$$

Combining Equations (1–3) and (8) yields:

(9) $$\begin{array}{l} \alpha =\;\text{arctan}\frac{m_{c}\;\text{tan}(f(\sqrt{m_{c}^{2}+n_{c}^{2}}))}{\sqrt{m_{c}^{2}+n_{c}^{2}}}\\ \beta =\;\text{arctan}\frac{n_{c}\;\text{tan}(f(\sqrt{m_{c}^{2}+n_{c}^{2}}))}{\sqrt{m_{c}^{2}+n_{c}^{2}}} \end{array}$$

Equations (7–9) illustrate the process to calculate the sun angles (*α, β*) from the sun spot centroid location (*m_c_, n_c_*) in the sun sensor body coordinates.

## 3. APS CMOS Detector Characteristics [6,15]

The STAR1000 is a CMOS image sensor with 1,024 by 1,024 pixels on a 15 mm pitch. It features on-chip Fixed Pattern Noise (FPN) correction, a programmable gain amplifier, and a 10-bit Analog-to-Digital Converter (ADC). All circuits are designed using the radiation tolerant design rules for CMOS image sensors, to allow high tolerance against to total dose effects, for more details about this one can refer to [5]. It is different from the CCD read out mechanism, for the APS based image sensors, in that registers that are directly accessed by the external controller contain the X- and Y-addresses of the pixels to be read. This architecture provides flexible operation and allows different operation modes such as (multiple) windowing, subsampling, and so on. Compared with the CCD detector, APS CMOS is more suitable as a detector for a sun sensor.

## 4. MEANSHIFT-Based High-Precision Positioning and Tracking Algorithm

The traditional spot positioning method is the correlation algorithm [5], but this approach has two shortcomings: (1) positioning accuracy is not high, and typically only 0.03° accuracy is achieved (2). It cannot track the movement of the sun spot. If the sun spot location changes with the satellite attitude, the correlation algorithm cannot track the movement of the sun spot and needs to renew the location of the sun spot, which is very complicated and time consuming. In this paper we employ FMMS which not only offers high positioning accuracy but also can be adaptive to track the location of spot movement.

### 4.1. The Spot Features Description and Similarity Function

In this paper we locate the coordinates of the centroid by identifying the spot, so we need to define the feature of the spot before identification. {*x_i_*}_*i* = *1*…*n*_ is a region of the normalized data points set (which could be viewed as pixels) with 0 as the center. An isotropic, convex profile with a monotonically decreasing kernel function *k(x)* weighted for all points, farther away from the center, the smaller the weight.

**Figure 4.**
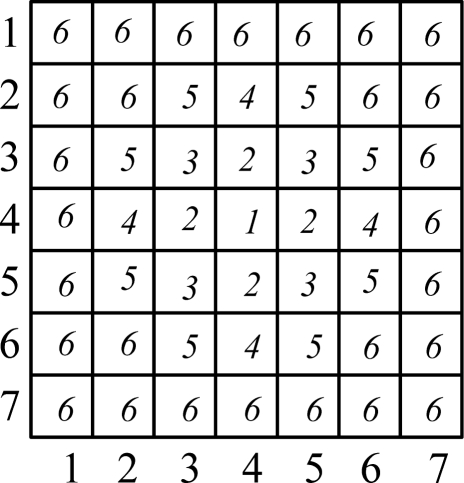
The quantified spot template.

**Figure 5.**
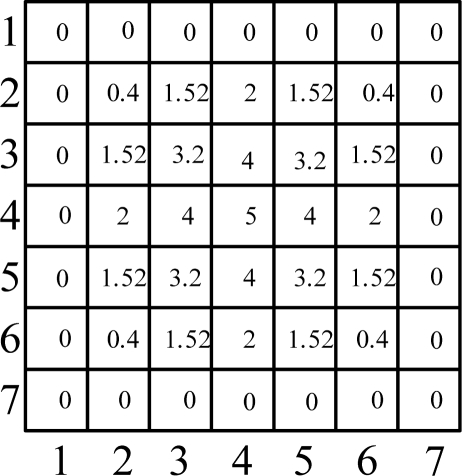
Kernel function weight distribution.

**Figure 6.**
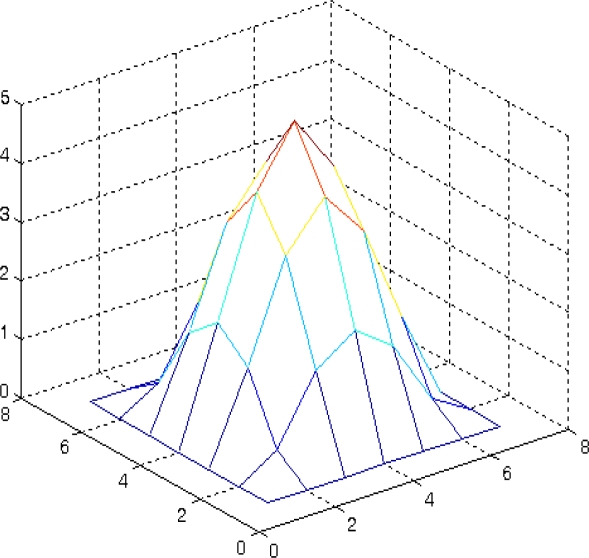
3-dimensional map of kernel function.

According to the MEANSHIFT [7] algorithm, calculating the spot features is divided into the following three steps:

1. Characterization of the pixel value (Figure 4). Defined function *b : R*^2^ → {1...*m*}, for each pixel, 0 ≤ *x* ≤ 255,*b(x)* is the quantified number of the pixels value in the quantitative feature space:
  (10) $$b(x)=\{\begin{array}{cc} 1 & x<t_{1}\\ 2 & t_{1}\leq x<t_{2}\\ \vdots & \vdots \\ m & t_{n}\leq x \end{array}$$
2. Select kernel function (Figures 5 and 6). There are many kinds of kernel function mentioned in [7], such as Epanechnikove kernel:
  (11) $$K_{E}\left(x\right)=\{\begin{array}{ll} C_{k}\left(1-{\left\|x\right\|}^{2}\right) & \left\|x\right\|<1\\ 0\;\;\;\;\;\;\left\|x\right\|\geq 1 & \end{array}$$
  normal kernel:
  (12) $$K_{N}\left(x\right)={\left(2\pi \right)}^{-d/2}\;\text{exp}\left(-\frac{1}{2}{\left\|x\right\|}^{2}\right)$$

In this paper we employed the Epanechnikove kernel. For the definition of the kernel function one can refer to [7], as no details are provided here.

3. Using the kernel function weighted all the quantified pixel, Calculated the spot feature vector as follows:
  $${\overset{⌢}{q}}_{u}=C\sum_{i=1}^{n}k_{E}\left({\left\|x_{i}\right\|}^{2}\right)\delta \left[b\left(x_{i}\right)-u\right],\;u=1\cdots m$$

In which constant C is a normalization function, makes the $\sum_{u=1}^{m}{\overset{⌢}{q}}_{u}=1$, then $C=\frac{1}{\sum_{i=1}^{n}k_{E}({\left\|x_{i}^{*}\right\|}^{2})}$.

**Figure 7.**
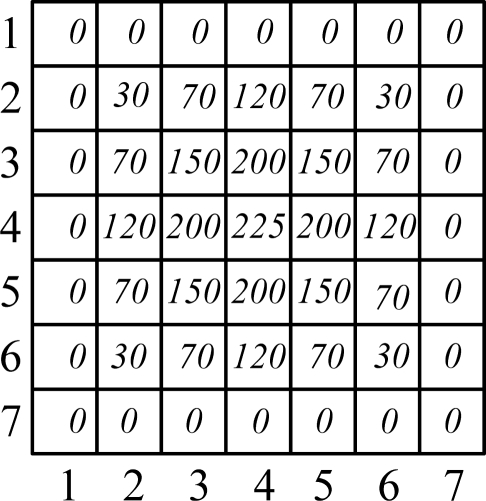
The simulated spot gray distribution.

For a simulated sun spot such as Figure 7. The quantified spot template is shown as Figure 5. The kernel function weight distribution is shown as Figure 6 and a 3-dimensional map of the kernel function is shown as Figure 7. The feature vector is:

*q*_1_ = 0.08999 *q*_2_ = 0.28798 *q*_3_ = 0.23038 *q*_4_ = 0.14399 *q*_5_ = 0.21886 *q*_6_ = 0.02880
*q̑* = [0.08999, 0.28798, 0.23038, 0.14399, 0.21886, 0.02880]

We set the candidate target model {*x_i_*}_*i*=1…*n_h_*_, with *y* as the center. Using the same kernel profile, the radius *h*, features *u* = *1…m* probability of occurrence in the candidate target model is:

(13) $${\overset{⌢}{p}}_{u}(y)=C_{h}\sum_{i=1}^{n_{h}}k_{E}({\left\|\frac{y-x_{i}}{h}\right\|}^{2})\delta [b(x_{i})-u]$$

If *C_h_* is a normalization factor, making $\sum_{u=1}^{m}{\overset{⌢}{p}}_{u}=1$, then:

$$C_{h}=\frac{1}{\sum_{i=1}^{n}k({\left\|\frac{y-x_{i}}{h}\right\|}^{2})}$$

The term *h* determines the scale of the candidate target. *q̑_u_* and *p̑_u_* (*y*) is the description of the feature of the goal and the candidate. Using the Bhattacharyya coefficient:

(14) $$\overset{⌢}{\rho }(y)\equiv \rho [\overset{⌢}{p}(y),\overset{⌢}{q}]=\sum_{u=1}^{m}\sqrt{{\overset{⌢}{p}}_{u}(y){\overset{⌢}{q}}_{u}}$$

we describe the degree of similarity between the feature of the goal and the candidate; the greater the coefficient, the more similarity between the goal and the candidate. The distance between them is defined as follows:

(15) $$d(y)=\sqrt{1-\rho [\overset{⌢}{p}(y),\overset{⌢}{q}]}$$

### 4.2. The Sun Spot Pointing and Tracking

With a description of the goal and the candidate target, and the criteria which can gauge their similarity degree, pointing and tracking the spot becomes a problem of searching for a new spot location in the current frame which made it possible that the function with *y* as independent variables could obtain the minimum distance. Searching begins from the previous location of a spot, and looking around in the neighborhood. Minimizing Equation (15) is equivalent to maximizing Equation (14). Assuming *y*_1_ is the spot location of the previous frame, we first calculate {*p̂_u_* (*y*_1_)}_*u*=1,…*m*_ in the current frame, if we expand the *ρ*[*p̑*(*y*), *q̑*] in the predicted location of *y*_1_, we then get the linear approximation of *ρ*[*p̑*(*y*), *q̑*]:

(16) $$\rho [\overset{⌢}{p}(y),\overset{⌢}{q}]\approx \frac{1}{2}\sum_{u=1}^{m}\sqrt{{\overset{⌢}{p}}_{u}({\overset{⌢}{y}}_{1}){\overset{⌢}{q}}_{u}}+\frac{1}{2}\sum_{u=1}^{m}{\overset{⌢}{p}}_{u}(y)\sqrt{\frac{{\overset{⌢}{q}}_{u}}{{\overset{⌢}{p}}_{u}({\overset{⌢}{y}}_{1})}}$$

This kind of approximation is satisfactory when there is little difference between the candidate {*p̂_u_* (*y*)} _*u*=1,…*m*_ and the initial {*p̂_u_* (*ŷ*_1_)} _*u*=1,…*m*_. In general, this assumption for the adjacent two frames is reasonable.

Combining Equation (13) with (16) yields:

(17) $$\rho [\overset{⌢}{p}(y),\overset{⌢}{q}]\approx \frac{1}{2}\sum_{u=1}^{m}\sqrt{{\overset{⌢}{p}}_{u}({\overset{⌢}{y}}_{1}){\overset{⌢}{q}}_{u}}+\frac{C_{h}}{2}\sum_{i=1}^{n_{h}}w_{i}k^{\prime }({\left\|\frac{y-x_{i}}{h}\right\|}^{2})$$

in which:

(18) $$w_{i}=\sum_{u=1}^{m}\delta [b(x_{i})-u]\sqrt{\frac{{\overset{⌢}{q}}_{u}}{{\overset{⌢}{p}}_{u}({\overset{⌢}{y}}_{1})}}$$

In this way, minimizing *d*(*y*) becomes to maximize the second half of Equation (17), which denotes the kernel density estimation calculated by using *k*(*x*) at the *y_1_* in current frame, so that we can use the MEANSHIFT procedure to find the great density estimation value (mode) in the neighborhood. In this process, the kernel shift from the current location *y* to the new location *y_1_*:

(19) $$y_{1}=\frac{\sum_{i=1}^{n_{h}}x_{i}w_{i}g({\left\|\frac{y-x_{i}}{h}\right\|}^{2})}{\sum_{i=1}^{n_{h}}w_{i}g({\left\|\frac{y-x_{i}}{h}\right\|}^{2})}$$

in which *g*(*x*)*=−k′*(*x*)

If we set the spot with a feature vector {*q_u_*}_u = 1,…,m_ at *y_0_* in the previous frame, *y*_1_ is the new spot location. Then the flow of the MEANSHIFT algorithm is:

Set the spot with a feature vector{*q_u_*}_u = 1,…,m_, at *y_0_* in the previous frame.

1. Calculate the feature vector of candidate spot {*p̑_u_* (*y̑*_0_)} _*u*=1…*m*_ of the first spot in current frame, and Bhattacharyya coefficient:
  $$\rho [\overset{⌢}{p}({\overset{⌢}{y}}_{0}),\overset{⌢}{q}]=\sum_{u=1}^{m}\sqrt{{\overset{⌢}{p}}_{u}({\hat{y}}_{0}){\overset{⌢}{q}}_{u}}$$
2. Calculate{*w_i_*}*_i=1…m_* with Equation (18).
  $$w_{i}=\sum_{u=1}^{m}\delta [b(x_{i})-u]\sqrt{\frac{{\overset{⌢}{q}}_{u}}{{\overset{⌢}{p}}_{u}({\overset{⌢}{y}}_{1})}}$$
3. Calculate the new location of spot with Equation (19)
  $${\overset{⌢}{y}}_{1}=\frac{\sum_{i=1}^{n_{h}}x_{i}w_{i}g({\left\|\frac{\overset{⌢}{y}-x_{i}}{h}\right\|}^{2})}{\sum_{i=1}^{n_{h}}w_{i}g({\left\|\frac{\overset{⌢}{y}-x_{i}}{h}\right\|}^{2})}$$
4. $\rho [\overset{⌢}{p}({\overset{⌢}{y}}_{1}),\overset{⌢}{q}]=\sum_{u=1}^{m}\sqrt{{\overset{⌢}{p}}_{u}(y_{1}){\overset{⌢}{q}}_{u}}$.
5. If *ρ*[*p̑*(*y̑*_1_), *q̑*] < *ρ*[*p̑*(*y̑*_0_), *q̑*], then ${\overset{⌢}{y}}_{1}\leftarrow \frac{1}{2}({\overset{⌢}{y}}_{0}+{\overset{⌢}{y}}_{1})$ until *ρ*[*p̑*(*y̑*_1_), *q̑*] > *ρ*[*p̑*(*y̑*_0_), *q̑*]
6. If ‖*y̑*_1_ – *y̑*_0_‖< *ɛ* then stop iteration to get the spot new location.
  $$\text{else}\;{\overset{⌢}{y}}_{0}\leftarrow {\overset{⌢}{y}}_{1}\;\text{jump}\;\text{to}\;2$$
7. Repeat steps 1–6 to get the coordinates of other sun spots.
8. Calculate the sun incident angle (*α_i_, β_i_*) *i* = 1…36.
9. Then the final sun incident angle (*α, β*) are
  $$\alpha =\frac{\sum_{i=1}^{n}\alpha_{i}}{n},\beta =\frac{\sum_{i=1}^{n}\beta_{i}}{n}$$

### 4.3. Over-Relax Technology Based Multi-Point Fast MEAN Shift Algorithm

For a single spot using MEANSHIFT algorithm positioning usually reaches sub-pixel accuracy, and in order to achieve high-precision positioning we need to use spot arrays. For each spot in the array we can get a pair of sun incident angles *α_i_, β_i_,* then the final sun incident angle is:

(20) $$\alpha =\frac{\sum_{i=1}^{n}\alpha_{i}}{n},\beta =\frac{\sum_{i=1}^{n}\beta_{i}}{n}$$

in which *n* is the number of the aperture.

As the MEANSHIFT computing process is complex, a multiple point MEANSHIFT procedure requires a lot of time, so in order to improve the real-time system, we need to study the fast MEANSHIFT algorithm. In this paper we use the over-relaxing method [8] to adjust the step size, and acceleration can be achieved. The over-relaxed bound optimization iteration is:

(21) $$y^{\left(k+1\right)}=y^{\left(k\right)}+\beta \cdot T_{\mathrm{step}}$$

In which *T_step_* is the MEANSHIFT vector. Apparently when the learning rate *β* = 1, over-relaxed optimization reduces to the standard MEANSHIFT algorithm. It is easily seen that when *β* > 1 acceleration is realized. However by simply assigning a fixed value to *β*, no convergence is secured and it seems quite difficult to get the optimal value for *β*. Xu *et al.* [9] proves that in the case of the Gaussian Mixture Model (GMM) parameter estimation, convergence can be guaranteed using this method when we are close to a local maximum and 0 < *β* < 2.This conclusion is generalized to the case of general bound optimization methods in [10,11]. Based on this important proposition, a simple adaptive over-relaxed bound optimization is readily available: the learning rate can be adjusted by evaluating the cost function. When the cost function becomes worse for some *β* > 1, then *β* has been set too large and needs to be reduced. By simply setting *β* = 1 immediately, convergence can be achieved. In this paper by regarding MEANSHIFT as a special case of bound optimization we presented the accelerated MEANSHIFT algorithm obtained in this way:

1. Initialization:
  Set the iteration index *k* = 1, and the step parameter *α* > 1.
2. Iterate until convergence condition is met:
  1. Calculate *ŷ*_*i*+1_ with Equation (19). And the MEANSHIFT vector
    m_G_ (*ŷ*_*i*+1_) = *ŷ*_*i*+1_ − *ŷ*_*i*_.
  2. *y*_*i*+1_ = *y_i_* + *β* m_G_ (*ŷ_i_*_+1_)
  3. If *ρ*(*y*_*i*+1_), > *ρ*(*y*_*i*_),
    Accept *y*_*i*+1_ and *β* = 1, *β* = *α* · *β*
    Else reject *y*_*i*+1_, and *y*_*i*+1_ = *ŷ*_*i*+1_, *β* = 1.
  4. Set *k=k+1*, start a new iteration
  5. If m_G_ (y_*i*+1_) < *ɛ* stop iteration.

## 5. The Test and Experiment

### 5.1. The Lab Test Equipment for an APS Sun Sensor

**Figure 8.**
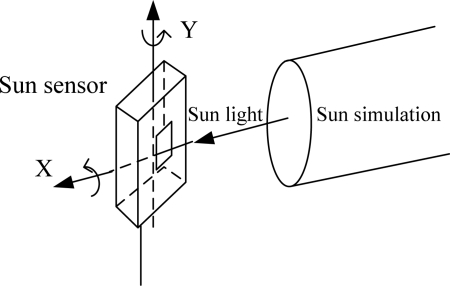
The lab test system for a sun sensor.

**Figure 9.**
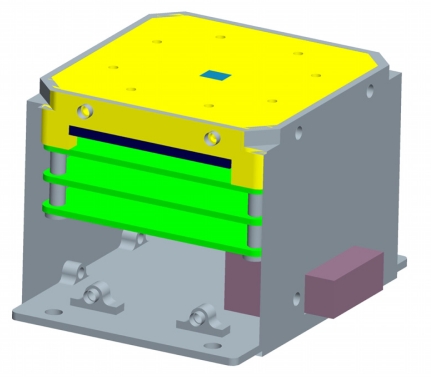
The diagram of an APS sun sensor.

The equipment used to test and calibrate the sun sensor consists of a sun simulator which can send a bundle of sunlight, a two-axis gimbals rotary table and a theodolite goniometer. The sun simulator has an angular diameter of 32 arcmin and a brightness of 0.1 sun constant. The sun sensor was fixed on the two-axis rotary table. The theodolite goniometer guarantees that the axis of sun sensor is collinear with the sun simulator’s. Arbitrary sun incidence angles can be established by rotating the table. The lab test system is shown in Figure 8. The diagram of an APS sun sensor is shown in Figure 9.

**Figure 10.**
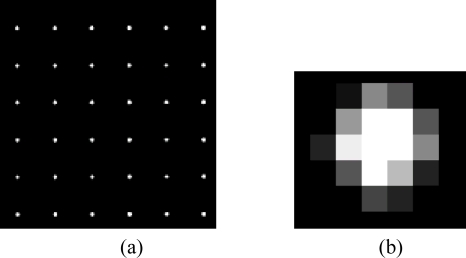
**(a)** Sun spots array image. **(b)** Single sun spot image.

The sun spot templates extracted from the sun image when the sun incident angles are both equal to 0. This work is carried out on the lab test equipment. The sun spots array image and a typical single sun spot template are shown in Figure 10(a,b). For each sun spot, we can get a template with 5 × 5 pixels.

### 5.2. Random Noise [12]

The sun sensor was placed on the two-axis turntable for about 1,000 seconds. A single aperture in the mask was chosen from the image with coordinates (*x,y*). The x and y values of the centroid is a function of time. The standard deviation on the x coordinate is 0.02 pixels and the standard deviation on the y coordinate is 0.02 pixels. Assuming a stable sun bundle, the coordinates of this aperture are supposed to be constant. The actual distance between the mask and detector is 4,000 μm, the size of APS CMOS pixel is 15 μm × 15 μm. One pixel therefore has an angle equivalent to arctan(15/4,000) = 0.21°. The random noise on a single aperture is then 0.02 × 0.21° = 0.0042° in each axis. This random noise is for a single aperture. Assuming that the measurements are independent, and the mask consists of 6 × 6 = 36 apertures, then the accuracy will be improved by sqrt (36) = six times. A noise estimate for this kind of sun sensor is then 0.0042°/6 = 0.0007°.

### 5.3. Experimental Parameter Setting

The choice of kernel function for the experimental results has a significant impact; this paper uses the following kernel function profile:

(19) $$K_{E}(x)=\left\{ \begin{array}{l} \begin{array}{cc} C_{k}(1-{\left\|\frac{x}{z}\right\|}^{2}) & \|\frac{x}{z}\|<1 \end{array}\\ 0\;\;\;\;\;\;\|\frac{x}{z}\|\geq 1 \end{array}\right.$$

where *z* denotes effective distance which is decided by the size of the sun spot. The term *x* actually represents the distance between the effective pixels and the center spot. If *z* is set too large or too small it will affect the pointing and tracking performance. If *z* is set too large it increases the computation and affect the system's real-time; when *z* is set too small, making the effective pointing and tracking of the region smaller, not only is operational efficiency reduced, but it is difficult to ensure positioning accuracy. In this paper *z* is set at 6.

**Figure 11.**
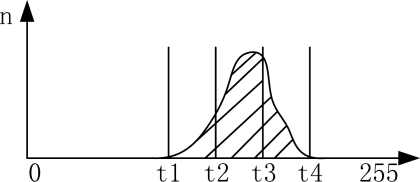
Choice of the feature value.

The election of the gray value of the pixel as a feature in general gray images; in this paper the gray value will be classified as follows (Figure 11): we choose four threshold values *t_1_,t_2_,t_3_,t_4_*. Five features are set: *q_1_ =* [*0,t_1_*)*, q_2_ =* [ *t_1_,t_2_*)*, q_3_ =* [ *t_2_,t_3_*)*, q_4_ =* [ *t_3_,t_4_*)*, q_5_ =* [ *t_4_,t_5_*)*,* in which *t_1_,t_2_,t_3_,t_4_* were set as 60,110,160,210, respectively.

According to the general considerations, the number of holes was designed to be 36 in this paper which is explained in Section 2, so that the the point accuracy of the sun spot array image location could reach a 0.01 ∼ 0.02 pixel level.

### 5.4. Calibration and Analysis

#### 5.4.1. Calibration

First of all, the sun sensor was fixed on the two-axis turntable, ensuring that the optical axis of the sun sensor was parallel with the sun simulator’s, controlling the turntable rotation around the x axis in steps of 1° from −64° to +64°, while keeping the y axis unrotated, then the turntable angle and the sun sensor angle can be recorded:

Turntable x axis angle: *X*[*x*_0_ *x*_1_ ⋯ *x*_128_]
Sun sensor angle: *α*[*α*_0_ *α*_1_ ⋯ *α*_128_]

Then we control the turntable rotatation around the y axis and keep the x axis unrotated to get the turntable y axis angle *Y*[*y*_0_ *y*_1_ ⋯ *y*_128_] and sun sensor angle *β*[*β*_0_ *β*_1_ ⋯ *β*_128_].

**Figure 12.**
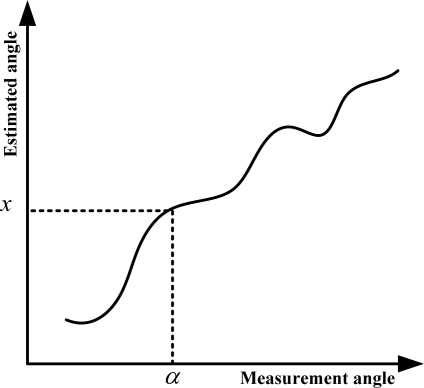
Relationship between the measurement angle and estimated angle.

Using MATLAB fitplot function (*x*, *α*, 6), the fitplot function (*y*, *β*, 6) is used to fit the two sets of data:

$$\begin{array}{c} x_{0}=A_{0}\alpha_{0}^{6}+A_{1}\alpha_{0}^{5}+A_{2}\alpha_{0}^{4}+A_{3}\alpha_{0}^{3}+A_{4}\alpha_{0}^{2}+A_{5}\alpha_{0}^{1}+A_{6}\\ x_{1}=A_{0}\alpha_{1}^{6}+A_{1}\alpha_{1}^{5}+A_{2}\alpha_{1}^{4}+A_{3}\alpha_{1}^{3}+A_{4}\alpha_{1}^{2}+A_{5}\alpha_{1}^{1}+A_{6}\\ \;\;\;\;\;\;\;\;\;\;\;\;\;\;\;\;\;\;\;\;\;\;\;\;\;\;\;\;\;\;\;\;\;\;\;\;\;\;\;\;\;\;\;\;\;\;\;\;\;\;\;\;\;\;\;\;\;\;\;\;\;\;\;\;\;\;\;\;\;\;\;\;\;\;\;\;\;\;\;\;\;\;\;\;\;\;\;\;\vdots \\ x_{128}=A_{0}\alpha_{128}^{6}+A_{1}\alpha_{128}^{5}+A_{2}\alpha_{128}^{4}+A_{3}\alpha_{128}^{3}+A_{4}\alpha_{128}^{2}+A_{5}\alpha_{128}^{1}+A_{6}\\ y_{0}=B_{0}\beta_{0}^{6}+B_{1}\beta_{0}^{5}+B_{2}\beta_{0}^{4}+B_{3}\beta_{0}^{3}+B_{4}\beta_{0}^{2}+B_{5}\beta_{0}^{1}+B_{6}\\ y_{1}=B_{0}\beta_{1}^{6}+B_{1}\beta_{1}^{5}+B_{2}\beta_{1}^{4}+B_{3}\beta_{1}^{3}+B_{4}\beta_{1}^{2}+B_{5}\beta_{1}^{1}+B_{6}\\ \;\;\;\;\;\;\;\;\;\;\;\;\;\;\;\;\;\;\;\;\;\;\;\;\;\;\;\;\;\;\;\;\;\;\;\;\;\;\;\;\;\;\;\;\;\;\;\;\;\;\;\;\;\;\;\;\;\;\;\;\;\;\;\;\;\;\;\;\;\;\;\;\;\;\;\;\;\;\;\;\;\;\;\;\;\;\;\;\vdots \\ y_{128}=B_{0}\beta_{128}^{6}+B_{1}\beta_{128}^{5}+B_{2}\beta_{128}^{4}+B_{3}\beta_{128}^{3}+B_{4}\beta_{128}^{2}+B_{5}\beta_{128}^{1}+B_{6} \end{array}$$

to get the two vectors: *A*(*A*_0_, *A*_1_ ⋯ *A*_6_), *B*(*B*_0_, *B*_1_ ⋯ *B*_6_), then we can get the relationship between the measurement angle and the estimated angle (Figure 12).

**Figure 13.**
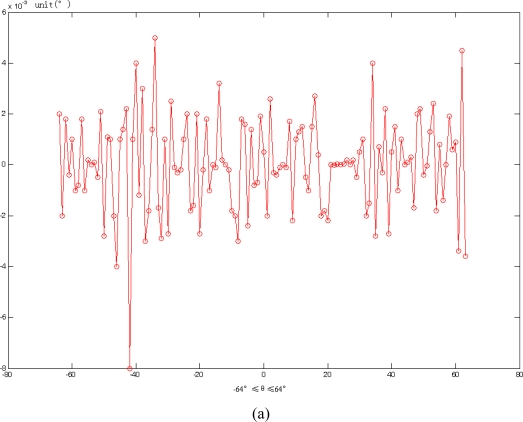

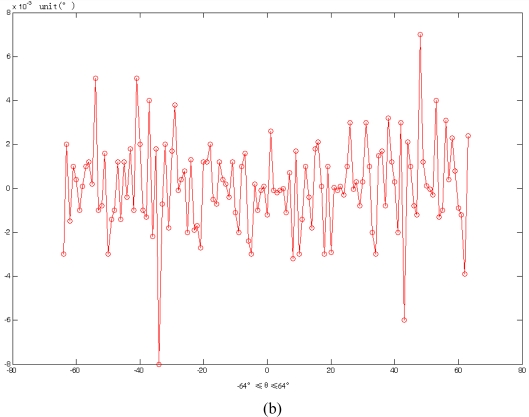
The accuracy of a sun sensor with incident angle in the range of −64° to +64°. **(a)** x axis result, **(b)** y axis result.

A high performance Fixed-Point Digital Signal Processor TMS320C6203 which was developed by the Texas Instruments Company was employed in the sun sensor for signal processing. The data update rate is 4 Hz. The final sun sensor accuracy is: X axis: 0.0063°, Y axis: 0.0068° (Figure 13).

#### 5.4.2. The Hole Block Detection Logic and Its Impact on the Accuracy

In space, some sun spots may be missing due to space debris, mask pollution or detector aging, *etc.* The multi-aperture sun sensor based on the FMMS algorithm has the advantage of maintaining a high accuracy even if some sunspots are missing. As the distance between the spots is relatively fixed, it can predict the location of holes in a certain effective area. When a similarity coefficient is less than a certain threshold in MEANSHIFT algorithm iteration process, the hole blocking. This method not only has high accuracy and the computation load is small, but it also does not affect system real-time performance.

**Figure 14.**
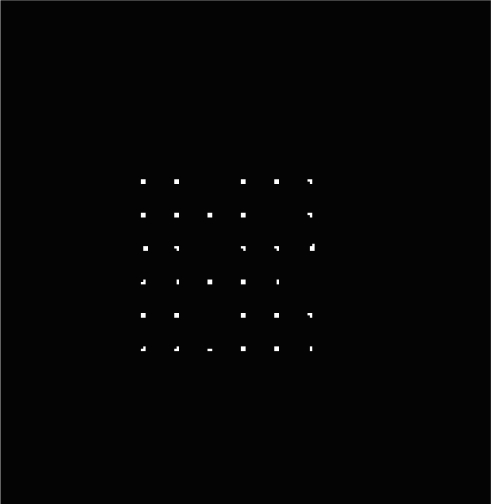
The image with one–five missing sun spots.

**Figure 15.**
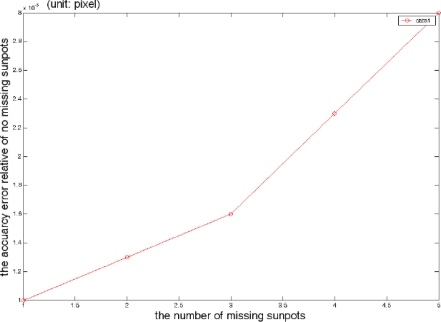
Accuracy analyses with 1–5 sun spots missing.

**Figure 16.**
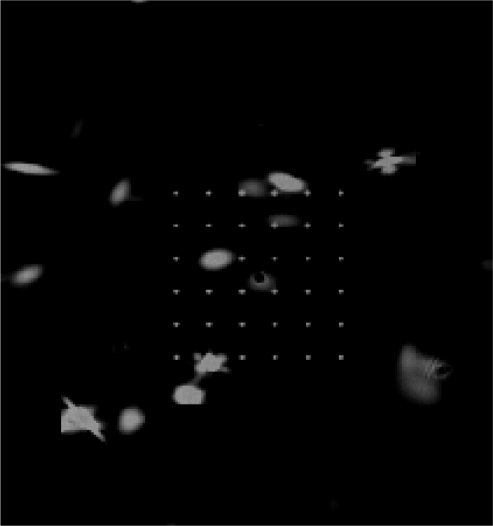
The simulated deteriorated image.

A typical sun spot array image with five spots missing is shown in Figure 14. An experiment is carried out to compare the accuracy deterioration under the conditions that one-five sun spots are missing relative to that of no sunspot missed. The experimental data are shown in Figure 15. From Figure 15, we concluded that the average accuracy error is no more than 0.003 pixels, which has almost no impact on the system accuracy. This immunity from missing sun spots significantly improves the adaptability for a harsh space environment, and this immunity cannot be achieved by a conventional single-aperture sun sensor. The conventional single-aperture sun sensor cannot distinguish between the sun spot and noise or bad pixels, while a multi-aperture sun sensor can overcome this drawback and extend the lifetime, since both the FMMS algorithms extract and identify the sun image based on the apertures array pattern, and the immunity to the partial sun spots missing or deterioration keeps the sun sensor away from a fatal fault. Figure 16 shows a simulated deteriorated image.

## 6. Conclusions and Future Work

In this paper, based on multi-aperture, APS detector flexible imaging and read out characters and a novel fast multi-point MEANSHIFT (FMMS) algorithm are proposed to improve the accuracy and reliability, the two key performance features of an APS sun sensor. The experimental results show that based on FMMS algorithm the multi-aperture the APS sun sensor presents many advantages compared with the traditional single-aperture sun sensor, particularly in high accuracy and reliability. With this system, the centroid accuracy of the sun image can reach to 0.01 pixels, even when some missing apertures and bad pixels appear on the detector because of aging of the devices and a harsh space environment, while the pointing accuracy of the single-aperture sun sensor using the conventional correlation algorithm is only 0.05 pixels.

When the sun incidence angle is greater than 45°, the sun spot is larger, the shape becomes elliptical, and sun sensor accuracy declined. In the next phase of our work we will study the reasons for this phenomenon and to find solutions to improve accuracy under wide sun angle conditions.
